# A Review on Indigenous Goats of East Africa: A Case for Conservation and Management

**DOI:** 10.3390/biology13060419

**Published:** 2024-06-05

**Authors:** Nelly Kichamu, Putri Kusuma Astuti, George Wanjala, Péter Strausz, Zoltán Bagi, Szilvia Kusza

**Affiliations:** 1 Centre for Agricultural Genomics and Biotechnology, University of Debrecen, Egyetem tér 1, H-4032 Debrecen, Hungary; kichamu.nelly@agr.unideb.hu (N.K.); astuti@agr.unideb.hu (P.K.A.); geog.wanjala@agr.unideb.hu (G.W.); bagiz@agr.unideb.hu (Z.B.); 2 Doctoral School of Animal Science, University of Debrecen, Böszözrményi út 138, H-4032 Debrecen, Hungary; 3 Institute of Animal Sciences and Wildlife Management, University of Szeged, Andrássy út 15., H-6800 Hódmezővásárhely, Hungary; 4 Department of Management, Institute of Strategy and Management, Corvinus University of Budapest, Fővám tér 8., H-1093 Budapest, Hungary; peter.strausz@uni-corvinus.hu

**Keywords:** East African goats, genetic characterization, indigenous goats, phenotypic characterization, production systems, population structure

## Abstract

**Simple Summary:**

Indigenous goats are important in the daily lives of people in East Africa because they adapt well to various climatic conditions. However, there is little information about these breeds both locally and internationally. This review discusses how these goats are raised, their physical and genetic characteristics, and diversity. It shows that there is no proper way by which these breeds are identified as most of them appear to be similar, hence they are named based on the region or tribe they are raised in. This review also points out several challenges like mixed breeding, lack of records, animal health problems, poor grazing fields, and basic care practices. To overcome these issues, it suggests the adoption of a participatory approach across goat value chains and the use of modern genomic tools in selection to improve goat breeding. This approach aims to protect the unique genetic resources of East African indigenous goats and ensure their future conservation.

**Abstract:**

Indigenous goats are important in the livelihoods of rural households in East African countries. This is due to their ability to produce and reproduce in different environments and climatic conditions. Even though these indigenous goats are important, there is little available information on the genetic characterization of these breeds in Africa and at the international level. This paper reviews the status of indigenous goats, highlighting their production systems, phenotypic and genetic characteristics, and genetic diversity, and proposes potential ways for sustainable improvement and conservation in East African countries. Most households use traditional production systems with various uncharacterized goat breeds and ecotypes, which are hence named after the tribe or locality in which they are found. Most of these goats are classified as small East African breeds, with significant variability in morphological features. Some of the challenges to goat production in this region are indiscriminate crossbreeding, lack of pedigree records, parasites and disease incidences, low-quality pastures, and low levels of management. There is a need for a collaborative approach amongst the actors in goat breeding value chains as well as integrating modern genomic tools into breeding programs to enhance selection. This will ensure the resilience and sustainability of these unique indigenous goat populations in East Africa

## 1. Introduction

Goats (*Capra hircus*) are recognized as one of the most ancient domesticated animal species in the world, with significant historical and cultural importance to human beings [1,2,3]. The wild goat (*Capra aegagrus*), commonly referred to as the bezoar, went through significant changes due to the process of domestication. Goat domestication began in the Fertile Crescent of Southeast Asia approximately 10,000 to 11,000 years ago, during the initial phase of human civilization [4,5,6]. However, recent archaeological and genetic studies argue that goat domestication started earlier than 10,000 years ago. For instance, [7] indicates that there is genetic evidence that goat domestication occurred earlier than the said 10,000 years ago, in the Zagros Mountains of western Iran, which shows that goats were among the first species to be domesticated after dogs. In addition, a study based on the large-scale mitochondrial DNA analysis of wild and domestic goats argues that the earliest domestication centers for the modern goat breeds were Eastern Anatolia and the Northern and Central Zagros Mountains [8]. Additionally, analysis of complete mitochondrial protein-encoding genes led to the discovery that the expansion of the wild goat population occurred during the late Pleistocene, indicating that goat domestication took place around 10,000 years ago [9]. Demographic analyses using the multiple sequentially Markovian coalescent method indicate that the divergence times between modern Asian and European goat populations might have predated the archaeologically estimated domestication time [10]. These findings suggest that goat domestication took place earlier than 10,000 years ago, with the Zagros Mountains and Eastern Anatolia as the primary domestication centers. These goats supplied early humans with essential commodities such as meat, milk, and hides. Factors like trade, conflict, and migration drove their domestication and subsequent global dispersion, spreading them to Europe, the Mediterranean, and finally East Africa via historical migration routes [11,12]. Today, there are over 576 recognized goat breeds worldwide, with 96 originating from Africa. This spread reflects an incredible array of adaptive features and benefits including meat and milk production as well as the fiber and mohair these goats provide [12,13,14].

Indigenous goats are defined as goat breeds that are native and adapted to the specific region or country in which they are produced [15,16]. These breeds can produce and reproduce well in these regions without stress. The breeds are regarded as the most important livestock resource in East African countries by providing employment and food. For instance, in Kenya, livestock contributes 12% of the overall gross domestic product (GDP) and approximately 40% of agriculture’s GDP, while employing almost half of the rural people [17]. In Tanzania, it accounts for about 7 to 8% of the gross domestic product (GDP) [18] and, in Uganda, it contributes 4 to 5% of the country’s gross domestic product [19]. Besides this, these breeds play a significant cultural role in various ceremonies and the social status of these communities as well as improving agricultural productivity through mixed farming systems and provision of essential resources such as manure [17].

Despite their adaptable characteristics and economic, social, and cultural importance, indigenous goats are often known to be underperforming in terms of production when compared to international breeds. The increase in demand for animal protein due to population growth in these countries [20] has led to the expansion of commercial livestock production. The introduction of international goat breeds has contributed to the ongoing erosion of indigenous goat genetic diversity in these regions. Moreover, the move towards more intensive and market-oriented livestock production systems has marginalized traditional pastoralist and smallholder production systems that had been the guardians of these indigenous goat genetic resources. Additionally, the movement of livestock between these neighboring countries in search of pasture and water especially during dry seasons leads to the intermixing of these indigenous breeds causing interbreeding amongst the different populations [21]. This scenario together with the cross-border livestock trade compromises the genetic potential of these breeds.

If this continues, the unique genetic resource will eventually become extinct. The current climate change and its impacts, which are characterized by the rise in temperatures, fluctuating rainfall patterns, extreme weather conditions, and increased CO_2_ levels [22], has continued to disrupt conventional livestock and farming practices, posing significant threats to global food security, particularly in Africa where nearly one in four people lack sufficient food for a healthy life [23]. In these circumstances, international goat breeds will not be able to provide for the livelihoods of the current and future population.

**Figure 1:**
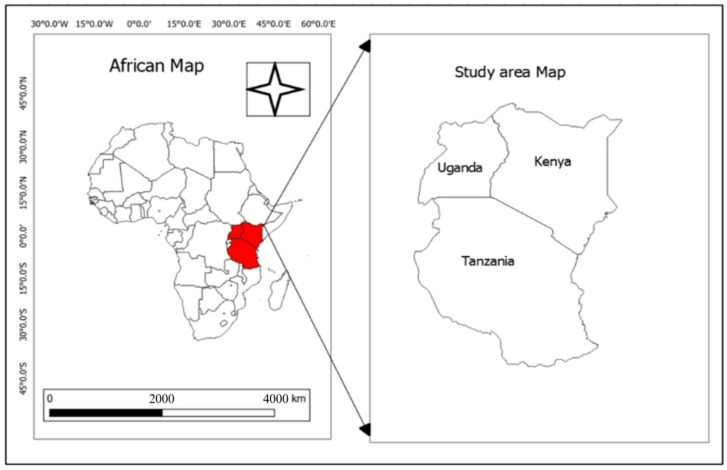
A map showing the study area.

For this reason, indigenous breeds capable of resisting these changing conditions will be needed above all. Therefore, conserving their distinct genetic background is critical for sustainable agricultural development and the resilience of future food systems. Therefore, this review aims to improve understanding and promote the use of indigenous goats in East Africa (Figure 1), providing an overview of their present status, focusing on their genetic diversity and production system. In addition, we examined the challenges facing the continued utilization of indigenous goats, analyzed their strengths and weaknesses, and developed a proposal which will help in improving the management and conservation of indigenous goats in East Africa.

**Figure 2:**
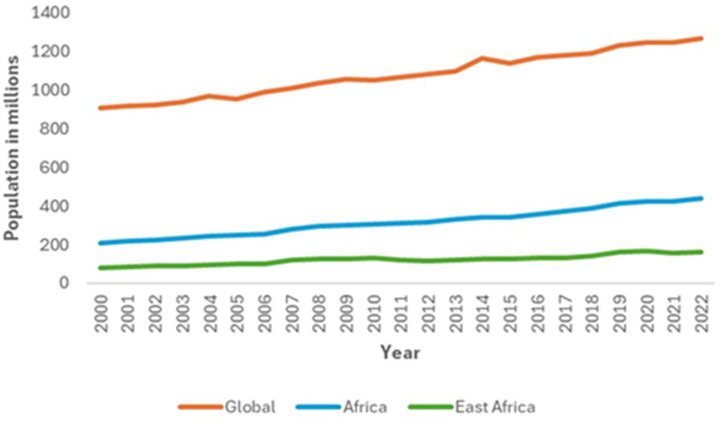
Trends in goat populations (Faostat, 2022).

According to [24], the global goat population has shown a steady increase to the current estimate of over 1269 million goats in 2022 from 907 million in 2000. The overall goat population for the African region has also shown an increasing trend, which reached its highest point in 2022. Africa accounts for approximately 34% (440 million) of the global goat population, while East Africa accounts for 36.6% (161 million) of the total goat population in Africa (Figure 2).

The increase in population explains the potential significance of these areas as key contributors to goat industry value chains and makes the case that goats are an integral part of global agriculture. Therefore, there is a need for sustainable production and management practices to safeguard the goats’ productivity.

## 2. Characteristics and Significance of Indigenous Goats

The majority of goats in East Africa are made up of indigenous breeds, accounting for approximately 95% of the goat population, and they are mostly bred by smallholder rural households and pastoral communities [25]. These indigenous breeds play a key role in improving the livelihoods of smallholder rural communities, by providing sustainable food, ensuring the community has enough income to cater for their needs, and being an important investment for this target population [26,27,28]. The resilience of these goats, which is shown by their ability to survive in challenging environmental conditions such as valleys and rocky and mountainous regions, is attributed to their distinctive conformation and adaptive traits, allowing them to survive in such environments [25,29,30]. Interestingly, the smaller body size and minimal nutritional requirements of these indigenous goats enable them to survive on constrained land resources, promoting their absorption into varied agricultural systems. They are thereby consistent with the needs of expanding population trends [31].

These indigenous goat breeds are known for their fast growth rates and prolificacy as most of them start breeding at an early age, often between eight months to one year, and often give rise to twins. For instance, studies on the topology and characterization of 11 indigenous goats in Tanzania found that among the studied does, the twinning rate was between 35 and 76.07% [32]. This prolificacy together with a short gestation period of about five months places them in a better position to get higher returns over a shorter period compared to other ruminants such as cattle [29,31,32,33]. In terms of nutrition, goat meat stands out for its numerous health advantages, characterized by its leanness and relatively low levels of fats, which makes it a preferred meat of choice for health-conscious consumers [34]. The nutritional benefits and socio-economic and environmental adaptation of these indigenous goats call for sustainable management and conservation of these breeds.

East Africa has several indigenous goat breeds and ecotypes. Unfortunately, most of these breeds cannot be distinguished at the phenotypic level. The lack of defined genotypes leaves local goat populations in East Africa identified through a confusing array of regional and tribe names as well as the colors and patterns of their coats. For instance, the Sukuma goat, which is named after the Sukuma tribe in Tanzania, and the Galla goat, which is named after the Galla tribe in Kenya [31,32,35]. In Tanzania, the common indigenous goat breeds include Ujiji, mostly found around Lake Tanganyika; Sukuma, found in the south of Lake Victoria; Maasai, Pare, and Sonjo in the northern region; while Gogo and Newala goats are found in the central and southern part of the country [32]. Indigenous goat breeds in Uganda include Mubende, Karamoja, Sebei, Teso, and Kigezi. These goats are distributed extensively in the northern and eastern savannah ecological areas and drier areas of Central Uganda [31]. In Kenya, the common indigenous goats are the small East African (SEA) and Galla. The Galla goats are found in arid and semi-arid parts of northern and north-eastern Kenya [36]. Although these goats have many names, research has shown significant genetic similarities across the breeds, showing that certain breeds may have distinct names but are part of the same breed ancestor.

While indigenous goats have various uses, they are mostly reared for meat production within subsistence agricultural systems, most of which are categorized as small East African goats. The term “Small East Africa” refers to animals with small body sizes, characterized by low birth weights, low milk yields, slow growth rates, and modest mature weights as compared to international breeds such as the Boer goat. For example, the small East African goat (SEA) in Kenya weighs between 1.2 and 1.8 kg at birth, and 25 kg at maturity, and reaches sexual maturity at four months while weighing 14–16 kg [37]. In Tanzania, mature SEA goats in traditional farming systems typically range from 20 to 30 kg for females and 28 to 35 kg for males, with low carcass weights of 10–12 kg when slaughtered at one year of age [38]. In Uganda, studies have found that the average mature weight of SEA goats is 26.1 and 27.4 kg for females and males, respectively [39], while the birth weights of Mubende and Teso goats were 1.98 and 1.54, respectively [40]. These low weights can be attributed to various factors, including insufficient intake and low-quality feed, disease outbreaks, exposure to harsh environmental conditions, and poor management as herders move for long distances in search of suitable pasture and water for these breeds, sometimes enduring entire days without access to water.

### 2.1. Phenotypic Characterization of Indigenous Goats in East Africa

Describing goats is an important process that can help farmers understand and analyze the different breeds and populations. This is achieved through breed characterization, which involves collecting all the important information and making accurate predictions about genetic performance in a given environment [41]. The Food and Agricultural Organization (FAO) has developed a global plan of action for animal genetic resources, which highlights the importance of farmers having a detailed understanding of breed characteristics to make informed decisions in livestock development and breeding initiatives [42]. In East Africa, indigenous goats display a wide range of morphological differences, including variations in coat color patterns, horn orientations, and ear sizes (Table 1). For example, plain, patchy, and spotted coat color patterns are prevalent in these breeds, and there are differences in horn orientation as some breeds have straight horns facing backwards (Gogo and Pare), while others have curved horns (Maasai and Galla). Additionally, variations are seen in facial structure, with some breeds having a straight face (Pare and Sukuma) and others having a concave face (Sonjo and Gogo). Most indigenous goats generally have medium and horizontal ears, with a few having large and floppy ears (Galla).

### 2.2. Genomic Adaptation of Indigenous Goats in East Africa

The impact of climate change is a global concern that has negatively affected the whole agricultural system. The global average temperature is expected to increase by 2 °C and will continue to increase by 0.15 °C per decade in the coming years [44]. In Africa, the average temperatures are expected to increase by 0.2–0.5 °C per decade [45], while in East Africa this temperature is projected to increase by between 1.6 and 1.9 °C by 2030, and between 2.7 and 3.9 °C by 2080 [46]. This change in global temperature will result in heat stress in both crops and livestock, negatively affecting livestock productivity. In addition, extreme temperatures have been linked with increases in disease incidences and deaths of livestock, and are hence likely to compromise the ability of animals to resist infections and produce [47]. For instance, the increase in temperatures in some regions of East African countries (Kenya and Tanzania) between 1991 and 2009 foresaw the massive loss of various livestock species, especially during the 2009 drought that killed from 70% to 90% of livestock in the pastoral Maasai community [48]. These deaths affected livelihoods and the country’s economy at large (Table S1).

In this respect, the expected rise in average temperatures and the rising demand for livestock products, particularly those from small ruminants, indicate that there is a need to find sustainable solutions to ensure livestock productivity in the face of these changes. Several studies have found genomic regions that are important in imparting adaptive features in indigenous goats. These features help them to survive and produce in different environmental situations (Table S1). These genes show signature selection, through which indigenous goats adapt to their environments while adjusting their physiological and biological functions to deal with challenging conditions. Understanding this genetic background not only sheds light on their evolutionary history but also provides significant markers for selective breeding programs targeted at enhancing goat population resilience and productivity. Therefore, it is hypothesized that the indigenous goat population may have distinct genes responsible for this adaptation. For instance, the HSPA2 gene variations in indigenous goats have been linked to improved heat tolerance, allowing these breeds to support homeostasis and productivity in high-temperature situations [35]. In addition, studies on the genome-wide characterization of selection signatures and runs of homozygosity in Ugandan goat breeds have also reported the presence of IL10RB and IL23A genes responsible for the immune system [31]. These are important genes for survival in contexts with limited veterinary services in the country. MAPK3 genes are important for cell growth and metabolism in response to nutrients, energy levels, growth factors, and survival [49]. The HSP70 gene also found in some goats is critical for the thermoregulatory role of the testes where spermatogenesis occurs. These genes, found in some indigenous goats, have been related to enhanced resilience to high temperatures which is critical for survival in contexts of frequent droughts [50].

### 2.3. Genetic Diversity Indices of Indigenous Goats in East Africa

Genetic characterization is considered essential for genetic improvement and conservation programs of farm animal genetic resources. However, the genetic diversity status of many African indigenous goats has not been sufficiently defined. Their breed composition has been the subject of much discussion up to now. The situation may have been created by inconsistent policies, limited use of advanced genomic technologies, and incomplete representation of African breed genomes in genotyping tools, which hinders molecular characterization efforts in the continent [51,52]. There are a few studies on the genetic characterization of African indigenous goats which indicate a high genetic diversity, while others show uniformity in genetic structure due to the sharing of related ancestors (minimal population substructure). Despite the large number of indigenous goat breeds and ecotypes in East Africa, only a few breeds with few samples that are not representative of the country goat population have been analyzed for their genetic characteristics using the current genomic tool, the caprine bead chip.

To address this, there is a need for an enhanced genetic characterization of the East African goat breed to help support a sustainable breeding method in the face of climate change and limited genetic knowledge. In Table 2, the observed heterozygosity (*H_o_*) varied from 0.459 (Ujiji goat in Tanzania) to 0.66 (Gogo breed in Tanzania), while the expected heterozygosity (*He*) ranged from 0.470 (Sebei breed in Uganda) to 0.68 (Gogo breed in Tanzania). It is important to acknowledge that these findings were obtained using microsatellite DNA markers and studies were carried out at different points in time, some of which were five years ago. The genetic condition of indigenous goat breeds in Africa may have undergone considerable changes now. High heterozygosity is crucial for the evolution of species, and it is important to make every effort to preserve it. Across these three studies, a consistent trend appeared, with indigenous goat populations generally showing moderate to high levels of genetic variation and relatively low levels of inbreeding. This implies that there is considerable potential for further improvement through selective breeding within these populations, contributing to their genetic vigor and diversity.

## 3. Common Indigenous Goat Production Systems in East Africa

**Figure 3:**
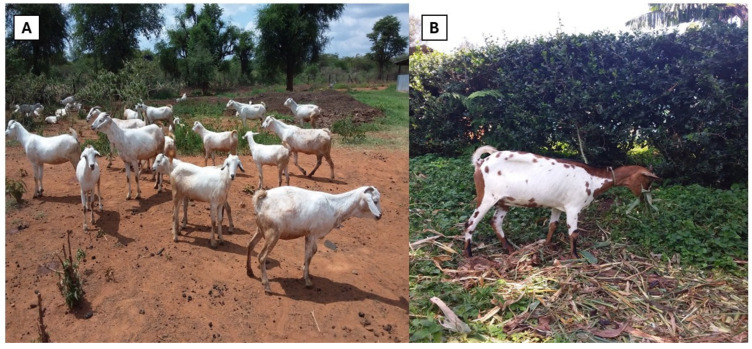
(**A**) Extensive/pastoral production and (**B**) mixed cropping livestock production systems.

A livestock production system refers to the method used in animal husbandry [54]. In East Africa, there are various livestock production systems used with goats (Table S3), but the commonly used ones are the mixed crop–livestock production system and the extensive/pastoral system [55,56] as presented in Figure 3. These systems are preferred because a bigger percentage of indigenous goats are found in the arid and semiarid regions of these countries and are often raised alongside other livestock species such as cattle and sheep. The production of these indigenous goats does not require a lot of investment costs and is carried out by small-scale farmers. However, there are some challenges encountered in these systems, including the small size of grazing fields as more of the grazing land is being used for human settlement and the little that remains has to be shared among livestock and crop production. Additionally, drought is another problem as it leads to feed scarcity and a lack of clean water [25,31,32].

### 3.1. Mixed Crop–Livestock Production System

A mixed production system is a popular farming approach where crops and livestock are raised together on the same piece of land. The system benefits both the animals and crops, as the livestock provide manure, while they also receive help from the remains of crops after harvesting. Integrating crops and livestock helps to improve nutrient recycling thus reducing the use of inorganic fertilizers, which results in sustainable agricultural practices and a reduction in the environmental footprint. This farming method is commonly used by small-scale farmers who keep goats, along with other livestock such as sheep and cattle, and contributes to about 70% of their household income [57]. Goats are preferred over other animals because they complement cattle and sheep, rather than competing with them for feed. This is because goats can eat a wider variety of vegetation. These goat herds usually graze freely on communal pastures and sometimes on fallow crop fields. The goats do not get proper health care and no extra supplementations are provided [25]. However, due to the increasing population pressure in such areas, free grazing is becoming limited. As a result, goats are now tethered, reflecting the challenge of getting sufficient feed in this system.

### 3.2. Pastoral Production System/Extensive

The extensive livestock production system, also referred to as the pastoral system, is widely practiced in East African countries. A large proportion of the population in these countries are pastoralists. This pastoral/extensive system plays an important role in the economy of this people. For example, in Tanzania, pastoralism is practiced by about 20% of the population and contributes 80% to the country’s livestock sector, making a significant contribution to foreign exchange earnings [57]. Similarly, in Kenya, pastoralism is estimated to be the basis of livelihood for 10% of the population [58]. The Maasai, Boran, and Rendile are some of the East African pastoralist societies that face increasing demands on their traditional way of life, posing challenges to the sustainability of the pastoral production system [59]. Therefore, the pastoral production system, with its various challenges and contributions, is still a crucial aspect of the agricultural and economic landscape in Kenya, Tanzania, and Uganda.

This system allows animals to graze freely on rangelands, fields of harvested crops, woodlands, and even the sides of roads. Sometimes, a shepherd keeps watch over them as they browse on shrubs. This extensive production system encourages the unrestricted movement of goats, enabling communal intermingling during herding which can lead to disease outbreaks [60,61]. Goats are often deprived of permanent night shelter; this exposes them to outdoor sleeping conditions thus compromising their welfare. In addition, it is difficult to understand the extent to which the animals in this production system have met their daily nutritional requirements through their dietary intake, as their grazing behavior on grasslands, residual crop materials, and sporadic browsing on shrubs with no supplementation cannot be assessed, which ultimately contributes to low productivity in these breeds [62].

The system also exposes the animals to unplanned breeding because it is challenging to regulate mating in such a situation, as males and females typically graze beside each other during the day, and in certain places, they are confined to one place at night [63]. Additionally, because there is no set mating season and it is difficult to tell which doe was served and which buck was used, it encourages inbreeding within the flock [64]. High mortality rates are also a concern because uncontrolled breeding encourages animals to conceive and even give birth when there is inadequate or lack of feed. Premature birth, dystocia, giving birth to poor kids, and, to a lesser extent, increased kid mortality rate are all made more likely by uncontrolled breeding practices [31]. This places a financial burden on breeders, as they must distribute a significant number of resources to address these issues.

### 3.3. Challenges Facing Indigenous Goat Genetic Resources in East Africa

Indigenous goats in East African countries are mainly raised under a traditional management system, relying on rangelands and harvested crop fields for feed [31]. However, they face several challenges including droughts and irregular rainfall patterns, which lead to water and feed shortages for livestock, causing economic losses and food insecurity in the affected regions. Providing veterinary care and controlling the spread of diseases among these breeds can be difficult due to remote and extensive grazing areas, which can result in potential disease outbreaks [65]. For instance, a study conducted by [66] revealed that gastrointestinal parasites are a major concern for goat farmers in Uganda under the free-range system. Additionally, studies by [67] found that 100% of goat samples studied in some African countries were found to be infested by gastrointestinal nematodes.

This arises when animals are grazed in a communal field which allows extensive mixing of herds from different regions resulting in a high incidence of diseases and parasite spread. In addition, the lack of proper health care has also contributed to high mortalities in these breeds as there are limited or no extension services offered to these farmers. To address this, farmers are forced to treat their animals on their own without understanding which disease they are treating or what dosage to administer. This leads to the build-up of drug resistance, an increase in morbidity and mortality rates, abortions, or subclinical symptoms such as low weight and poor production [39]. This ultimately affects animal welfare and the country’s economy as well. Competition for the available resources, such as water and pasture, is another common challenge in indigenous goat production, as everyone wants to have enough grazing area and water for the animals. This later brings about conflicts among pastoralists and the neighbouring agricultural communities [32].

Encroachment of wildlife on pastoral lands can also result in conflicts, especially when livestock are predated upon, or crops are damaged [68]. In communal areas, inbreeding and uncontrolled mating are common issues, as mentioned above, which arise due to the lack of planned selection programs and organized breeding seasons. This results in poor productivity, as does and bucks graze together and are confined under the same roof throughout the year. The absence of an organized genetic improvement program with the challenging nature of production systems for extensive and smallholder goat farmers complicates the identification and recording of important traits, such as disease resistance, nematode resistance, and reproduction efficiency.

**Figure 4:**
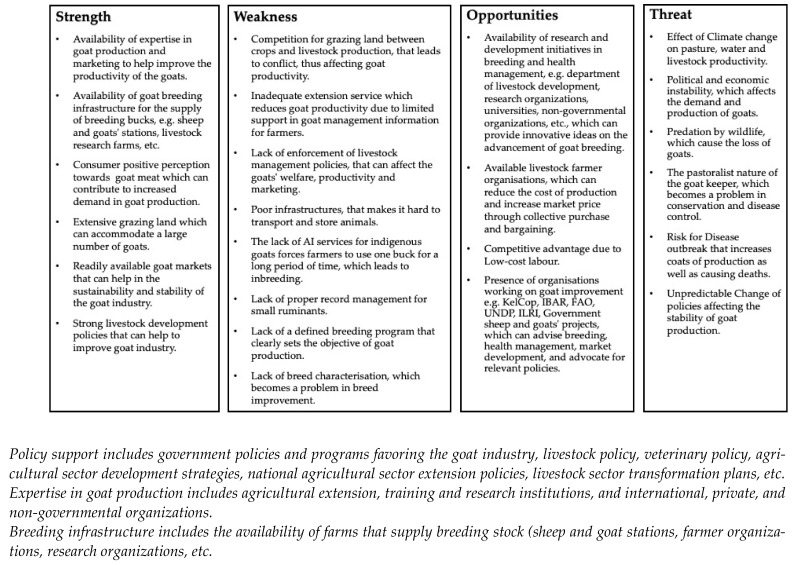
SWOT analysis for indigenous goats in East Africa.

For this reason, it is advisable to do a deeper investigation into the challenges faced by these production systems and come up with breeding goals that will ensure the development of a sustainable genetic improvement program and safeguard the genetic resources of indigenous goats in East Africa [69]. To address this challenge, there is a need to address the activities that will ensure sustainable land and resource management practices, improved infrastructure, access to agricultural education and veterinary services, and develop and enforce the favorable policies that can support the resilience and adaptation of pastoral and agro-pastoral communities to changing conditions and develop a breeding program that will improve important traits such as production, functional traits, and disease resistance. Figure 4 shows the SWOT analysis of indigenous goats in East Africa.

## 4. Case Studies, Proposed Conservation, and Management Intervention Program for Indigenous Goats

Improving goat breeds for better productivity and adaptability is important for the region’s economies. This can be achieved by increasing productivity while also ensuring their genetic makeup is not compromised. Artificial insemination technologies have been used in some countries to address this. For example, the use of low-cost artificial insemination (AI) in Ethiopia has helped enhance national economic growth and the genetic diversity of small ruminants. Besides this, the introduction of breeding buck stations in the country has helped conserve and improve indigenous breeds. In this method, the selected bucks are allowed to stay at a particular station where communities can access them easily [70]. To prevent inbreeding in this system, the bucks are only allowed to stay at one station for less than 24 months. This system also allows the rotation of bucks through careful coordination with a breed association.

**Figure 5:**
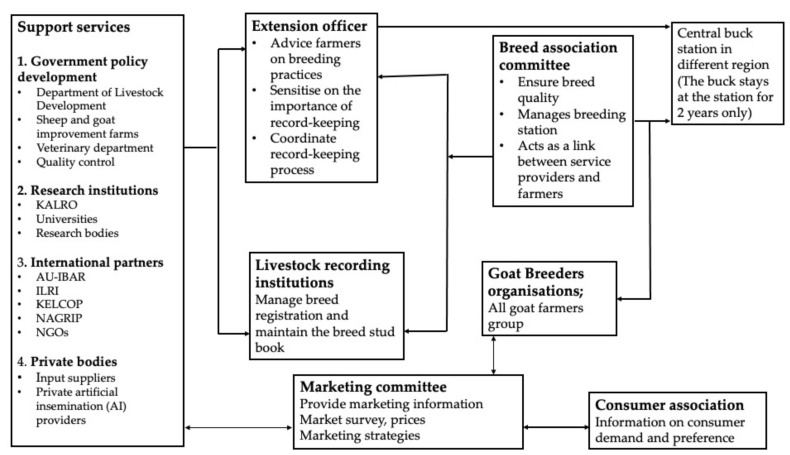
Proposed framework for conservation and management of indigenous goats.

The formation of breed associations or farmer organizations has played a bigger role in ensuring the smooth running of buck stations. For example, the formation of community-based and farmer-led goat improvement programs in the Nyeri and Meru regions of Kenya has proven a notable success. These programs, under the facilitation of non-governmental organizations (GTZ) and the Integrated Small Livestock Project (ISLP) in Nyeri and FARM-Africa in Meru, have helped the formation of the Dairy Goat Association of Kenya (DGAK) and the Meru Goat Breeders Association (MGBA), respectively. The same model was also initiated by the Farm Africa Goat Improvement Project (FA-GP) in Tanzania. These initiatives have been so effective that, within less than seven years, there has been an improvement in the dairy goat population. Additionally, through these associations, farmers have increasingly adopted record-keeping for individual animals and are using these records to decide the market price for their breeding animals [71,72,73]. The above method can also be adopted for the conservation of indigenous goats in East Africa. Figure 5 shows a proposed framework that can be adopted to safeguard the indigenous goats of East Africa.

To ensure the diffusion and adoption of this framework to farmers, there is a need to have consistent and reliable extension services or agents to help in the sensitization and coordination of these important strategies. This can be enhanced by ensuring that there is a strong collaboration between farmers, extension officers, research scientists and governments. To conserve these indigenous goats, it is important to develop relevant policies that will be favorable in promoting their conservation and sustainable management. This involves setting up well-designed breeding programs, well-specified breeding goals and activities to address improved feeding, health management practices, and breeding activities, and a sustainable recording system for individual animal production and performance data.

Furthermore, the use of molecular and genomic tools has been seen to be more effective in detecting and mapping genes of economic importance in goats such as disease resistance, functional traits, and adaptation. Most goat studies using microsatellites have revealed high genetic diversity and adaptive genes in East African goat breeds [26,31,53]. Hence, the use of next-generation sequencing and genotyping technologies such as the Illumina Goat SNP70K bead chip may provide opportunities for genomic breeding schemes in future. Such important suggestions will not only protect these goat breeds but also address the challenges faced by farmers. In addition, it will help in developing a planned breeding program which can help in identifying traits of interest for selection, ultimately enhancing the resilience and productivity of indigenous goat populations in East Africa.

## 5. Discussion

This review assessed the status of indigenous goats in East Africa. These goats are important livestock in Kenya, Tanzania, and Uganda, contributing significantly to the gross domestic product (GDP), economic stability, and social and cultural welfare of the countries [25,26,27,28]. The ability of these breeds to produce and reproduce in unfavorable environments and their low-cost production [25,29,30] make them valuable genetic resources in these countries’ rural, arid, and semi-arid regions. The presence of unique genetic traits such as heat tolerance and disease resistance in these goats is vital for enhancing livestock resilience against climate change [31,35,48]. Despite this, the genetic characterization and understanding of these goat populations are insufficient, and this calls for more genomic studies that will guide in developing a targeted breeding program for functional and disease resistance traits. This includes the use of various molecular markers such as restriction fragment length polymorphism (RFLP), randomly amplified polymorphic DNA (RAPD), amplified fragment length polymorphism (AFLP), microsatellite typing, single nucleotide polymorphism (SNP), and whole genome sequencing [74]. While these goats present various advantages, they face several challenges, including environmental problems such as drought, disease outbreaks [65], and overgrazing [32]. These environmental changes come with various problems including heat stress on animals, inadequate feeds, and lack of clean water. These affect the welfare of the animals leading to reduced productivity and reproduction and even increased mortality. Socioeconomic issues such as land disputes and lack of access to veterinary services [68], which further worsen the vulnerability of these indigenous goat populations, are also problematic. For instance, land disputes can significantly impact goat productivity in that when communities engage in land disputes, it creates insecurity for the animals in grazing, leading to a reduction in the amount of land available for grazing. This, in turn, may affect the cost of production, as farmers have to purchase additional feed for their animals. In addition, land disputes may restrict farmers from accessing the market for their livestock and livestock products. This makes it hard for farmers to sell their animal products at a good price, hence lowering the value of goat enterprises. The lack of veterinary services on the other hand may force farmers to treat their animals without expert knowledge, posing a significant risk. This is because many of the drugs they use may provoke resistance or fail to treat the correct disease, leading to a high mortality rate and making farming unprofitable. These challenges require a coordinated approach that combines sustainable land management, improved veterinary care, and community-based conservation strategies as mentioned in the proposed program and case studies provided. Future research should focus on the genetic characterization of indigenous goat populations using advanced molecular markers to identify and conserve important adaptive traits. Furthermore, there is a need for relevant policies which are followed and enforced to support the sustainability of indigenous goats, including initiatives that encourage genetic conservation, improve livestock management practices, and enhance market access for smallholders. Networks with international organizations are also important for using scientific research and technology to improve goat production systems.

## 6. Conclusions

Indigenous goats are not only a resource for economic development in East African countries. They also form an important part of the strategies to address future sustainable development goals (SDGs). Given the pressures of climate change and population growth, there is an urgent need to implement these management and conservation interventions. Favorable policy support and continued research on indigenous goat studies should be encouraged, as they hold a chance of changing the traditional farming system into a more resilient, sustainable, and viable business in East Africa.

## Figures and Tables

**Table 1:** Phenotypic description of common indigenous goats in East Africa.

Breed	Country	Color	Coat Pattern	Horn Type	Ear Type	Hair Type	Face Type	Back Type	References
Gogo	Tanzania	White in color		Straight with backward orientation	Medium horizontal	Medium and smooth	Concave and straight	Straight	[32]
Pare		White	Pied white and red	Curved with backward orientation	Medium horizontal	Short and course	Straight	Straight	
Sonjo		Plain red	Spotted white and red	Curved with upward orientation	Small horizontal	Medium and course	Concave	Straight	[43]
Sukuma		Pied black and white	Pied white and red	Straight with backward orientation	Medium horizontal	Short and smooth	Straight	Straight	
Maasai		Plain white		Curved	Small and erect	Short		Straight	
Galla	Kenya	White	Some black and brown stripes	Curved	Medium and floppy	Smooth			[36]

**Table 2:** Genetic diversity of indigenous goats in East Africa.

Breed	Country	Observed Heterozygosity (Ho)	Expected Heterozygosity (He)	References
Gogo	Tanzania	0.660 ± 0.030	0.680 ± 0.130	[53]
Pare	Tanzania	0.590 ± 0.040	0.640 ± 0.090	
Sonjo	Tanzania	0.650 ± 0.030	0.650 ± 0.120	
Sukuma	Tanzania	0.580 ± 0.040	0.630 ± 0.160	
Mbeya	Tanzania	0.530 ± 0.025	0.541 ± 0.051	[26]
Maasai	Tanzania	0.527 ± 0.023	0.482 ± 0.066	
Ujiji	Tanzania	0.459 ± 0.024	0.473 ± 0.066	
Teso	Uganda	0.464 ± 0.022	0.515 ± 0.061	
Kigezi	Uganda	0.506 ± 0.025	0.509 ± 0.060	
Sebei	Uganda	0.559± 0.024	0.470 ± 0.064	[53]
Karamoja	Uganda	0.495 ± 0.025	0.525 ± 0.060	
Small East Africa	Kenya	0.477 ± 0.024	0.504 ± 0.068	
Galla	Kenya	0.485 ± 0.025	0.492 ± 0.063	

## Data Availability

Data are contained within the article and Supplementary Materials.

## Supplementary Materials

The following supporting information can be downloaded at: https://www.mdpi.com/article/10.3390/biology13060419/s1, Table S1. Effect of drought on livestock in some East African countries; Table S2. Genes for adaptation and immune response in some indigenous goats; Table S3. Various goat production systems in East Africa. References [25,31,33,35,49,50,51,54,75,76,77,78,79] are cited in the Supplementary Materials.
