# Supplementary material for: A Review on Indigenous Goats of East Africa: A Case for Conservation and Management
Source: Biology (Basel). 2024 Jun 5;13(6):419. doi: 10.3390/biology13060419 (PMC11200369; doi:10.3390/biology13060419)
Supplement: Supplementary file 1 [file biology-13-00419-s001.zip › biology-3022209-supplementary.pdf]

Table S1. Effect of drought on livestock in some East African countries [1].

Drought Period	Country/Region	Percentage loss in livestock
1991-1992	Northern Kenya	70% loss of livestock
1999-2001	Kenya	30% loss of cattle, 30% loss of sheep and goats, 18% loss of camels
2004-2006	Kenya	70% loss of livestock in pastoral regions
2009	Tanzania and Kenya	70 – 90% loss of livestock in the Maasai community

Table S2. Gene for adaptation and immune response in some indigenous goats

Breed	Country	Number	Method	Candidate gene	Responsibility	References
Mubende	Uganda	29	Illumina GoatSNP50 Bead Chip	PPP1R36	Heat shock protein.	[2]
				A2(HSPA2)	Heat stress	
SEA	Uganda	29	Illumina GoatSNP50 Bead Chip	ERBB2 &ENO1	Immunity regulation	[2]
Galla	Kenya	12	Illumina GoatSNP50 Bead Chip	HYAL1, HYAL3	Immunity regulation	[3,4]
				MST1, PCK	Adaptation	
Sebei	Uganda	15	Illumina GoatSNP50 Bead Chip	H0XC12, H0XC13	Thermoregulation	[2]
Karamoja	Uganda	29	Illumina GoatSNP50 Bead Chip	KPNA4 (CHI1), MTOR	Heat shock	[2]
Kigezi	Uganda	29	Illumina GoatSNP50 Bead Chip	IL10RB &IFNLR1	Immunity Regulation	[2]
Galla	Kenya	96	mtDNA D-loop and HSP70 gene	HSP70	Thermoregulatory role in the testes where spermatogenesis occurs	[5]
Begiat	Ethiopia	11	Whole-Genome Sequencing	SLC6A2, SLC7A11, SLC26A8, ATP6V1H,	For immune response and adaptation	[6]
				MAPK13, MAPK14, SCN7A, IL12A, EST1 DEFB116 & DEFB11		
Barki	Egypt	59	caprine and ovine 50K SNP BeadChips	FGF2, GNAI3, PLCB1	Thermotolerant	[7]
				MYH, TRHDE, ALDH1A3	Energy and digestive metabolism	
				GRIA1, IL2, IL7, IL21, IL1R1		
					Nervous and autoimmune response	
Aberegalle	Ethiopia	11	Whole-Genome Sequencing	PPP4R3B	Regulate lipid metabolism and energy homeostasis.	[8]
				UGT2A2	Regulate the metabolic pathways	

Table S3. Various goat production systems in East Africa

Category	Production system	Description	Output	Challenges	References
**Small scale**	Pastoral	Move from place to place in search of pasture and water.<br>Small population<br>Mixed species (goats, sheep and cattle), communal grazing, shared land, not confined at night	Low yields of meat and milk	Drought, Disease, conflict, uncontrolled breeding, inadequate veterinary services, high mortality rate, lack of record keeping, poor nutrition, low yield and poor-quality animals.	[2,9,10]
	Agro-pastoral	Settled with Small population.<br>Mixed crops and livestock, uncontrolled mating, open communal grazing, feed on crop residues	Low yields of meat and milk	Drought Disease, conflict, uncontrolled breeding, inadequate veterinary services, high mortality rate, lack of record keeping, poor nutrition, low yield and poor-quality animals.	[11]
	Mixed smallholder farming	Small number of flocks, feed on crop residues, kitchen wastes<br>Mixed crops and livestock<br>Small farms<br>Practice tethering of animals, uncontrolled mating	Low yields of meat and milk	Drought, Disease, conflict, uncontrolled breeding, inadequate veterinary services, high mortality rate, lack of record keeping, poor nutrition, low yield, and poor-quality animals.	[11]
	Urban and sub urban	Small herd size<br>In towns, feed on kitchen wastes, crop residues and commercial feeds.<br>Tethered or roam around the town.<br>Uncontrolled mating	Low yields of meat and milk	Drought, Disease, conflict, uncontrolled breeding, inadequate veterinary services, high mortality rate, lack of record keeping, poor nutrition, low yield and poor-quality animals	[12]
Large scale	Ranching	Big population of animals<br>Large farms<br>For business<br>Planned mating systems, access to veterinary services.<br>Established fodder, commercial feeds	High productivity	Fluctuating market prices<br>Disease outbreak, drought, high cost of feeds, intensive selection.	[13]
	Government /state owned	Big population, managed by the government.<br>Proper management, selection of quality does and bucks for breeding.<br>Controlled mating system.<br>Access to veterinary services.	High productivity	Fluctuating market prices<br>Disease outbreak, drought, high cost of feeds, high cost of breeding stock.	

References

1. Kumar, M.; Gupta, J.; Meena, H.R. *Sustainable Livelihood through Livestock Production Systems*; LAP Lambert Academic Publishing: Saarbrucken, Germany, 2019.

2. Onzima, R.B.; Upadhyay, M.R.; Doekes, H.P.; Brito, L.F.; Bosse, M.; Kanis, E.; Groenen, M.A.M.; Crooijmans, R.P.M.A. Genome-Wide characterization of Selection Signatures and Runs of Homozygosity in Ugandan Goat Breeds. *Front. Genet.* 2018, *9*, 318. https://doi.org/10.3389/fgene.2018.00318.

3. Waineina, R.W.; Okeno, T.O.; Ilatsia, E.D.; Ngeno, K. Selection signature analyses revealed genes associated with adaptation, production, and reproduction in selected goat breeds in Kenya. *Front. Genet.* 2022, *13*, 858923. https://doi.org/10.3389/fgene.2022.858923.

4. Kamidi, C.M.; Waineina, R.W.; Wasike, C.B.; Ilatsia, E.D.; Ngeno, K. Signature analysis of divergent selection revealed genes associated with different biological aspects of goats in Kenya. *Tanz. J. Agric. Sci.* 2023, *22*, 309–315.

5. Masila, E.M.; Ogada, S.O.; Ogali, I.N.; Kennedy, G.M.; Too, E.K.; Ommeh, C.S. Mitochondrial DNA D-Loop Polymorphisms among the Galla goats reveals multiple maternal origins with implication on the functional diversity of the HSP70 gene. *Genet. Res.*2024, *2024*, 5564596. https://doi.org/10.1155/2024/5564596.

6. Gebreselase, H.B.; Nigussie, H.; Wang, C.; Luo, C. Genetic Diversity, Population Structure and Selection Signature in Begait Goats Revealed by Whole-Genome Sequencing. *Animals* 2024, *14*, 307. https://doi.org/10.3390/ani14020307.

7. Kim, E.S.; Elbeltagy, A.R.; Aboul-Naga, A.M.; Rischkowsky, B.; Sayre, B.; Mwacharo, J.M.; Rothschild, M.F. Multiple genomic signatures of selection in goats and sheep indigenous to a hot arid environment. *Heredity* 2016, *116*, 255–264. https://doi.org/10.1038/hdy.2015.94.

8. Berihulay, H.; Li, Y.; Gebrekidan, B.; Gebreselassie, G.; Liu, X.; Jiang, L.; Ma, Y. Whole genome resequencing reveals selection signatures associated with important traits in Ethiopian indigenous goat populations. *Front. Genet.* 2019, *10*, 1190. https://doi.org/10.3389/fgene.2019.01190.

9. Kosgey, I.S.; Rowlands, G.J.; van Arendonk, J.A.M.; Baker, R.L. Small Ruminant Production in Smallholder and Pastoral/Extensive Farming Systems in Kenya. *Small Rumin. Res.* 2008, *77*, 11–24. https://doi.org/10.1016/j.smallrumres.2008.02.005.

10. Wilson, R.T. Goats and Sheep in the traditional livestock production systems in semi-arid Northern Africa: Their importance, productivity, and constraints on production. In *Livestock Development in Sub-Saharan Africa*; CRC Press: Boca Raton, FL, USA, 2019; pp. 91–106. https://doi.org/10.1201/9780429047008-8.

11. Asrat, G.A.; Yoseph, M.G.; Habtemariam, K. Integrating crop and livestock in smallholder production systems for food security and poverty reduction in Sub-saharan Africa. *Afr. J. Agric. Res.* 2018, *13*, 1272–1282. https://doi.org/10.5897/ajar2018.13020.

12. Alarcon, P.; Fèvre, E.M.; Muinde, P.; Murungi, M.K.; Kiambi, S.; Akoko, J.; Rushton, J. Urban livestock keeping in the city of Nairobi: Diversity of production systems, supply chains, and their disease management and risks. *Front. Vet. Sci.* 2017, *4*, 171. https://doi.org/10.3389/fvets.2017.00171.

13. Derner, J.D.; Hunt, L.; Filho, K.E.; Ritten, J.; Capper, J.; Han, G. Livestock production systems. In *Rangeland Systems*; Springer International Publishing: Cham, Switzerland, 2017; pp. 347–372. https://doi.org/10.1007/978-3-319-46709-2_10.
